# Profiling of Volatile Compounds and Associated Gene Expression and Enzyme Activity during Fruit Development in Two Cucumber Cultivars

**DOI:** 10.1371/journal.pone.0119444

**Published:** 2015-03-23

**Authors:** Shuxia Chen, Ranran Zhang, Lining Hao, Weifeng Chen, Siqiong Cheng

**Affiliations:** College of Horticulture, Northwest A&F University/Key Laboratory of Horticultural Plant Germplasm Resources Utilization in Northwest China, Yangling, Shaanxi, China; University of Malaga-Consejo Superior de Investigaciones Científicas, SPAIN

## Abstract

Changes in volatile content, as well as associated gene expression and enzyme activity in developing cucumber fruits were investigated in two *Cucumis sativus* L. lines (No. 26 and No. 14) that differ significantly in fruit flavor. Total volatile, six-carbon (C6) aldehyde, linolenic and linoleic acid content were higher during the early stages, whereas the nine-carbon (C9) aldehyde content was higher during the latter stages in both lines. Expression of *C*. *sativus* hydroperoxide lyase (*CsHPL*) mirrored 13-hydroperoxide lyase (13-HPL) enzyme activity in variety No. 26, whereas *CsHPL* expression was correlated with 9-hydroperoxide lyase (9-HPL) enzyme activity in cultivar No. 14. 13-HPL activity decreased significantly, while LOX (lipoxygenase) and 9-HPL activity increased along with fruit ripening in both lines, which accounted for the higher C6 and C9 aldehyde content at 0-6 day post anthesis (dpa) and 9-12 dpa, respectively. Volatile compounds from fruits at five developmental stages were analyzed by principal component analysis (PCA), and heatmaps of volatile content, gene expression and enzyme activity were constructed.

## Introduction

Cucumber (*Cucumis sativus* Linn.) is a widely consumed vegetable with a fresh and distinct flavor[1], and fruit quality is important for satisfying consumer demand [2]. The typical cucumber flavor results from the enzymatic action of LOX on linolenic and linoleic acids, which introduces molecular oxygen at C13 or C9, forming 13-hydroperoxylinolenic acid (13-HPOT) or 9-hydroperoxylinolenic acid (9-HPOT). HPL cleaves 13-hydroperoxide (13-HPO) and 9-HPO to produce the C6 and C9 aldehydes that are responsible for the cucumber flavor [3]. These aldehydes can then be reduced to the corresponding C6 alcohols by alcohol dehydrogenase (ADH). Studies have reported that only the oxylipin metabolic pathway contributes to aldehyde and alcohol content, and hence flavor [4].

To date, 78 volatile compounds have been identified in cucumber fruits, including aldehydes, alcohols, esters, alkanes, furfurans and others [5], and (*E*,*Z*)-2,6-nonadienal and (*E*)-2-nonenal are the main aroma compounds [6,7]. Besides aldehydes and alcohols, ketones and esters also contribute significantly to fruit flavor [5]. Hydroperoxide isomers of both linoleic and linolenic acids are produced by LOX, and HPL acts on these products to generate cis-C6 or C9 aldehydes and oxyacids [8]. C6 aldehydes and alcohols give cucumber its distinctive grassy, green aromas[9,10], while C9 aldehydes and alcohols contribute the typical flower-like qualities[11]. LOX and HPL are key enzymes in the oxylipin pathway that are encoded by multiple gene families, and Defilippi et al. (2009) reviewed the genes and enzymes responsible for fruit volatile formation[12]. A total of six *LOX* genes were identified in tomato [13–16], and *TomloxC* was shown to be involved in the production of C6 aldehydes and alcohols by antisense genetics approaches[13]. In kiwi, *AdLox3*, *AdLox4* and *AdLox6* are candidates for regulators of the synthesis of volatile compounds[17]. 23 *LOX* genes were identified in apple, in which *MdLOX1a* and *MdLOX5e* were identified as candidates for involvement in fruit aroma volatile production[18]. *HPL* gene family members involved in aroma volatile biosynthesis have been studied in cucumber fruit[19], melon [20], and grape[21]. Cultivar, development, ripening, environmental and storage conditions can all affect gene expression and enzyme activity of lipoxygenases and hydroperoxide lyase that influence volatile compound production [22,23]. The intensity of aromas is higher in intermediate- or late-season apricots, but lower in very-early-season fruits[24]. *LOX* expression, LOX and HPL activity, total antioxidant capacity, and hexanal production in two olive cultivars are influenced predominantly by genetic factors[25]. Volatile production can differ markedly in different cultivars, as can fruit development and ripening, both quantitatively and qualitatively. Understanding the genes and enzymes responsible for fruit volatile formation is important for determining the mechanisms of aldehyde formation and ultimately improving the quality of cucumber fruits.

In the past, research has mainly focused on the types of volatile compounds present in cucumber and their potential roles in fruit development. Ligor et al. (2008) reported that the volatile compounds in a cucumber cultivar from Poland were mainly aldehydes, alcohols, and ketones, with C6 and C9 aldehydes the main volatile compounds[26]. In the Chinese cucumber cultivar ‘Xintai mici’, both (*E*,*Z*)-2,6-nonadienal and (*E*)-2-nonenal increased during fruit development and peaked during ripening[27]. During cantaloupe fruit development, aldehydes were the main compounds, and levels increased as fruits matured [7]. The aroma threshold of several volatile compounds and their flavor characteristics have also been assessed [11,28]. Fruit flavor is largely correlated with the developmental stage, and there are large differences in the types and quantities of volatile compounds at different developmental stages. (*Z*)-3-hexenal and (*E*)-2-hexenal are responsible for the ‘green notes’ of *melon* fruits [7]. Changes in volatile compounds during fruit development have been studied in melon[29–31], watermelon[32,33], mango [34,35], and kiwi [2]. However, the relationships among gene expression, enzyme activity and fruit aldehyde formation in cucumber fruit development are not well understood [4,19,27,36].

In this work, we investigated the development of flavor in cucumber fruits by studying transcription of LOX/HPL pathway genes, the activity of associated enzymes, and the appearance of volatile products. Special emphasis was placed on the relationships among gene expression, enzyme activity, and aldehyde production during fruit development. C6 and C9 aldehydes had a particularly important affect on flavor during fruit development. Volatile compounds were detected by gas chromatography mass spectrometry (GC-MS), and linolenic and linoleic acids were determined by GC-FID. Data were analyzed by PCA to identify the main volatiles involved in the different developmental stages. These results provide a basis for further studies into the accumulation of C6 and C9 compounds in cucumber fruits.

## Materials and Methods

### Chemicals and reagents

Reference compounds were purchased from Sigma-Aldrich (Sigma-Aldrich, Shanghai). (E)-2-pentenal, (Z)-2-heptenal, and (6Z)-nonen-1-ol were purchased from Fluka (chromatographically pure, Shanghai, China); (E, E)-2, 4-heptadienal was purchased from Accustand (chromatographically pure, Shanghai, China). The standards used for fatty acid and organic acid analyses were obtained from Sigma-Aldrich (Sigma-Aldrich). Other chemicals, which were of analytical grade, were obtained from Sigma-Aldrich (Sigma-Aldrich).

### Plant material and sampling

The inbred line No. 26 used in this study has a relatively high 2,6-nonadienal/(*E*)-2-nonenal ratio (10.25) and an intense “cucumber-like” flavor. No. 14 has a lower 2,6-nonadienal / (*E*)-2-nonenal ratio (6.56). No. 26 and 14 are the parental lines used by our research group for breeding studies.

No. 26 was selected from high generation inbred lines of ‘Jinyan No.1’, a northern China ecotype and inbred line No. 14, which was selected from high generation inbred lines of ‘Deltastar’, which was a southern China ecotype. The two inbred lines were grown at the Horticulture College of Northwest A&F University during March-July, 2012. Row spacing was 60 cm; plant spacing was 20 cm. Three replicates were grown for each inbred line, and 80 cucumber plants per replicate were included.

Cucumber plants were grown in the greenhouse under natural sunlight, where minimum and maximum temperatures ranging between 12–14°C and 28–30°C, respectively. Opening female flowers at node 14 of uniform size were selected and marked and five fruits per replicate were collected at 9:00~10:00 o’clock in the morning at 0, 3, 6, 9, and 12 DPA and transported to the laboratory immediately in the icebox and screened for uniform size and freedom from visible defects or decay. The development stage and cultivar of female flowers was recorded as 26–0 or 14–0 for 0 DPA, 26–3 or 14–3 for 3 DPA, and so on. The fruits were divided into two groups, and the one group was analyzed for volatile compounds immediately and the other group were flash-frozen over liquid nitrogen and maintained at -80°C until analysis. Fruits were stored on ice immediately after thawing. Three fruits with three biological replicates were also used for biochemical and molecular analyses.

### Analysis of volatile compounds

Volatile compounds were measured as previously described [5] with slight modifications. For the analysis, 10 g of fresh cucumber flesh was ground over liquid nitrogen and transferred to a 10 mL vial. Sodium chloride (1 g, the concentration had better effect on the extraction basing on our previous experiment) and octanal (10 μL of a 1 μL·mL^-1^ solution, internal standard) were added, and vials were sealed and mixed using a vortex for 10 s.

For solid-phase microextraction (SPME), the extraction fiber was heated at 250°C for 2 h and transferred to the headspace of the vials. Volatile compounds were extracted in a 40°C water bath for 30 min and subsequently desorbed at 230°C for 3 min into the splitless injection port of a GC-MS (Thermo-Finnigan Trace 2000/Polaris Q GC/MS, Thermo Finnigan, China) fitted with an HP-INNWAX column (0.25 mm I.D. 60 m, 0.25 μm; Agilent, Shanghai, China). Chromatographic conditions consisted of an initial oven temperature of 40°C for 2.5 min, a final oven temperature of 230°C for 7 min, and a temperature increase rate of 6°C min^-1^. Nitrogen was used as the carrier gas at 1 cm s^-1^.

Volatile compounds were identified using the NIST 2002 mass spectra library (National Institute of Standards and Technology, USA), linear retention indices, literature data and mass spectra of authentic standards whenever possible. For quantification, characteristic quantifier ions were selected for each compound [37]. All samples were analyzed in triplicate.

### Analysis of fatty acids

Fatty acids were measured as previously described [38]with slight modifications. Fresh cucumber flesh was heated at 100°C for 3 min to inactivate enzymes and ground in liquid nitrogen. Ground sample (40 g) was transferred to a 250 mL flask containing 40 mL of chloroform and 80 mL of methanol. 20 μL of heptadecanoic acid (100 g L^-1^) was added as an internal standard, and the flask was sealed and mixed using a vortex for 15 s. The resulting precipitate was subjected to vacuum filtration and washed with 40 mL of chloroform. Approximately 40 mL of a 0.76% sodium chloride solution was added and the flask was mixed using a vortex for 30 min. The upper aqueous phase was discarded and the lipid phase was evaporated using a rotary evaporator.

To the lipid phase, 2 mL of 1:1 (v/v) petroleum ether-toluene and 2 mL of 1:1 (v/v) KOH-methanol were added, and the solution was mixed for 10 s and incubated at room temperature for 30 min. Distilled water (6 mL) was added and the solution was mixed for 10 s. The upper phase (2 mL) was subjected to gas chromatography (GC, Thermo/Finnigan Trace GC Ultra, Thermo Finnigan, Bremen, Germany) on a DB-WAX column (0.25 mm I.D. 30 m, 0.25 μm; Agilent), nitrogen was used as the carrier gas for gas chromatography (GC) equipped with A flame ionization detector (FID). The gas velocities were 1 mL min^-1^ for N_2_, 35 mL min^-1^ for H_2_, and 350 mL·min^-1^ for air. GC conditions consisted of an initial temperature of 180°C for 2.0 min, a temperature increase to 240°C at 8°C min-1, an injection temperature of 200°C, an injection volume of 1 μL, and a split ratio of 80:1.

### Measurement of LOX, HPL, and ADH activities

Fruits from 0, 3, 6, 9, and 12 dpa were collected, transported to the laboratory in ice boxes, and used immediately. The top, middle, and bottom cucumber fruits were separated, and 2–5 g of each was weighed and mixed. LOX activity was measured as previously described [3], as were HPL and alcohol dehydrogenase (ADH) activities [39]. The ADH assay reaction mixture contained 50 mM TES pH 7.5, 2.5 M NADH, and 0.125 mM acetaldehyde. 1 U of ADH was defined as the amount of enzyme required to decompose 1 mol of substrate per minute per g of protein.

### Expression of LOX and HPL

Total RNA was isolated from cucumber fruits using Trizol reagent (Tiandz, Beijing, Inc.) and treated with RNase-free DNase (Tiandz, Beijing, Inc) to remove genomic DNA. Reverse transcription was performed using approximately 1 μg of purified RNA as template with oligo(dT)_18_ and a Revert Aid First Strand cDNA Synthesis Kit (Fermentas) following the manufacturer’s instructions. *CsHPL* and *CsLOX* gene-specific primers for RT-qPCR were designed based on the *CsHPL* sequence (Accession No. AF229811) and *CsLOX* sequence (Accession No. KC429651), respectively. Quantitative PCR was performed using SYBR Green in a BioRad IQ5 PCR thermal cycler (Bio-Rad Co., USA). Real-time PCR conditions consisted of 95°C for 1 min, followed by followed by 40 cycles of 95°C for 15 s, 57°C for 30 s, and 72°C for 20 s. Actin cDNA was used as an internal control. The relative expression levels of LOX, HPL and other genes were calculated by the ΔΔCT method. PCR primers were designed using primer Premier 5.0 as follows; for *CsLOX*, *CsLOX*-RT F: 5′-GGA GAT GGT ACT GGA GAG CG-3′ and *CsLOX*-RT R: 5′-CAC GAC GAG GGT AAG GGA A-3′; for *CsHPL*, *CsHPL*-RT F: 5′-CTC CTT TCT CGC TTC TCA CC-3′ and *CsHPL*-RT R: 5′-C TCA AAC GAC ACG GCA TCA CT-3′.

### Statistical analysis

The data were tested for normality through Shapiro-Wilk test and the homogeneity of variances before the analysis of variance. All data are expressed as the mean ± SD of three replicates, and statistical analysis was performed using ANOVA within DPS v7.55 for Windows. Significant differences between fruits harvested at different developmental stages were confirmed using the Duncan's multiple range test. P-values <0.05 were considered significant. Principal component analysis (PCA) was used to detect clustering and to investigate possible relationships between different development stages and volatile compounds. To eliminate the influence of dimension, data were classified into 10 grades for PCA, that is, grade 1 < X - 2δ and grade 10 > X + 2δ, where the interval of every grade was 0.5δ, and δ was the standard deviation. Following peak annotation and normalization, the GC-MS data matrix after peak annotation and normalization was used for multivariate analysis with SPSS software version 13.0 (SPSS Inc., Chicago, IL, USA).

Hierarchical cluster analysis (HCA) and heatmap generation was carried out using Euclidean distance and Ward clustering algorithms to analyze the relationship between the volatile compounds, *CsHPL* gene expression, and C6, C9 and fatty acid content. The output from Cluster (http://rana.stanford.edu/software/) was submitted to TreeView (http://rana.stanford.edu/software/).

## Results and Discussion

### Changes in volatile compounds during cucumber fruit development

There were 21 aldehydes, 15 alcohols, and nine other volatile compounds identified in the cucumber cultivar No. 14 inbred line, and 23 aldehydes, 17 alcohols, and 10 other volatile compounds in the No. 26 cultivar. Volatile compounds were mainly aldehydes and alcohols, and most were similar in both cultivars. Particular volatile compounds were detected throughout cucumber fruit development, and these included 10 aldehydes and three alcohols in No. 14, and 8 aldehydes and three alcohols in No. 26.

### Compounds influencing cucumber fruit aroma

The effects of volatile compounds on cucumber flavor is dependent on the threshold content, which is the lowest concentration that can be perceived by the sense of smell in humans, and this gives rise to the aroma value, which is the ratio between volatile content and threshold content. Volatile compounds with aroma values >1 have the greatest effect on cucumber flavor, and the higher the aroma value, the greater the influence on flavor. In this study, 13 aroma impact compounds were identified: (*E*,*Z*)-2,6-nonadienal, (*E*)-2-nonenal, hexanal, (*E*)-2-hexenal, (*E*)-6-nonenal, nonanal, (*Z*)-2-heptenal, pentanal, propanal, (*E*,*E*)-2,4-heptadienal, 1-nonanol, 1-nexanol, and (*Z*)-3,6-nonadien-1-ol. The aroma threshold content of (*E*,*Z*)-2,6-nonadienal, which gave the lowest value, was 0.01 ng g^-1^, whereas the volatile content of (*E*,*Z*)-2,6-nonadienal, which exhibited the highest aroma value, was 0.093–1.018 μg g^-1^. (*E*)-6-nonenal had an aroma threshold content of 0.02 ng g^-1^, a volatile content of 0.033–0.122 μg g^-1^, and an aroma value of 1.65×10^3^–6.1×10^3^, which was lower than that of (*E*,*Z*)-2,6-nonadienal. (*E*)-2-nonenal had an aroma threshold content of 0.5 ng g^-1^, a volatile content of 0.024–0.132 μg g^-1^, and an aroma value of 48–264. These results revealed that there were significant differences in volatile content, and consequent differences in aroma values.

(*E*,*Z*)-2,6-nonadienal and (*E*)-2-nonenal were reported to be aroma impact compounds in cucumber fruits from the ‘SMR 58’ cultivar, along with hexanal, (*E*)-2-hexenal, propanal and (*E*)-6-nonene [40]. In this study, nonanal, (*Z*)-2-heptenal, pentanal, (*Z*)-3,6-nonadien-1-ol, (*E*,*E*)-2,4-heptadienal, 1-nonanol, and 1-hexanol were the main aroma impact compounds identified (Table 1). Flavor characteristics are known to be determined by the compounds with the highest aroma values [7,11]. Even so, compounds not designated as aroma impact compounds can contribute to fruit flavor, as shown previously in peach [41]. The flavor characteristics in cucumber were attributed to aroma impact compounds, comprising hydrocarbons, acids and alcohols.

**Table 1:** Aroma values, odor thresholds and volatile content of cucumber fruits.

Compound	Odor Threshold content (ng g^-1^)	Volatile content (μg g^-1^)	Aroma value	Flavor description
(*E*,*Z*)-2,6-nonadienal	0.01	0.093–1.018	9.3×10^3^–1.018×10^5^	Cucumber-like
(*E*)-2-nonenal	0.5	0.024–0.132	48–264	Tallowy
Hexanal	4.5	0.074–0.67	16.4–148.9	Green grass
(*E*)-2-hexenal	17	0.024–0.468	1.41–27.5	Apple like
(*E*)-6-nonenal	0.02	0.033–0.122	1.65×10^3^–6.1×10^3^	Fruit-like
Nonanal	1	0.002–0.003	2–3	Flower-like
(*Z*)-2-heptenal	0.8	0.004–0.029	0.5–36.25	Fresh
Pentanal	12–42	0–0.132	0–3.14	Fresh
Propanal	9.5–37	0.147–2.732	15.4–73.8	Stimulate
(*E*,*E*)-2,4-heptadienal	10	0.051–0.843	5.1–84.3	Fresh
1-nonanol	50	0.011–0.383	0.22–7.66	Rose
1-hexanol	250	0.019–1.338	0.076–5.35	Strawberry, fresh
*(Z*)-3,6-nonadien-1-ol	10	0.033–0.166	3.3–16.6	Musky

### Qualitative and quantitative changes in volatile compounds

**Fig 1:**
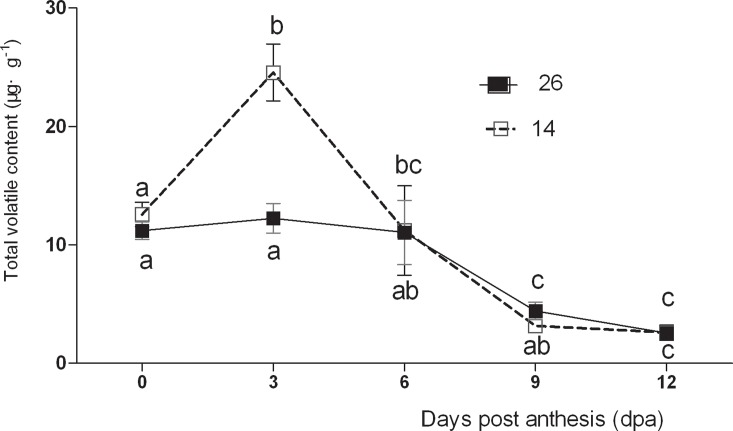
Changes in total volatile content during cucumber fruit development. The data are presented as means ± standard deviation, Data were analyzed by 1-way ANOVA followed by the Duncan’s multiple range tests to make comparisons within each line, values with different small letter mean significant difference (P<0.05), while with the same mean no significant difference (P>0.05). We have added the changes in the figure captions. The same was as below.

The total volatile content of fruits in this study was higher during the early stages of fruit development and decreased significantly after 6 dpa (Fig. 1). The total volatile content during the early stages were higher than those in the late (mature) stages of fruit development, and increased significantly by 3 dpa, at which point they were 195.47% higher than at 0 dpa. The total volatile content was lowest at 9–12 dpa. The changes in total volatile content were insignificant between 0–6 dpa in No. 26, but decreased markedly after 6 dpa, with levels at 9 dpa 38.97% higher than at 6 dpa.

**Fig 2:**
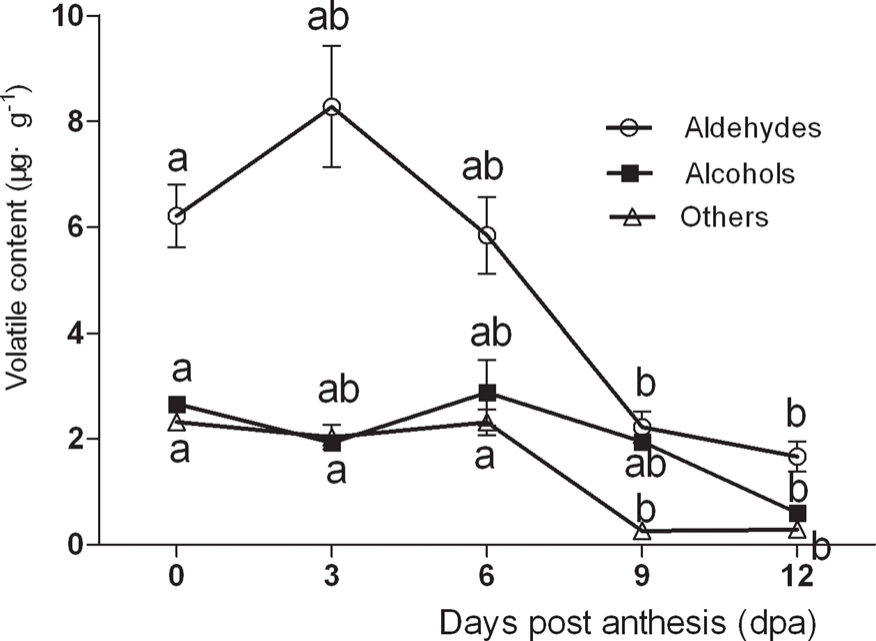
Changes in different types of volatile compounds in No. 14.

**Fig 3:**
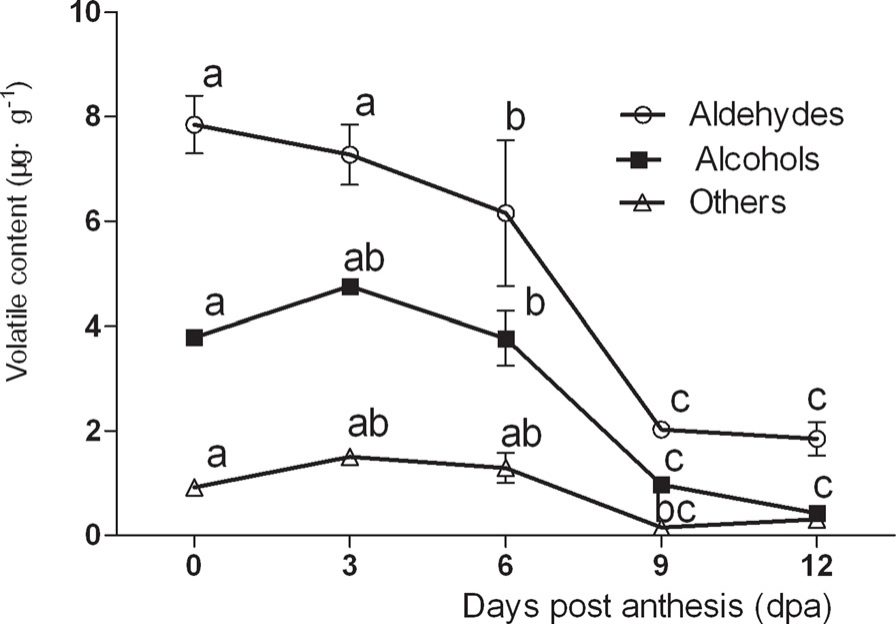
Changes in different types of volatile compounds in No. 26.

The types of volatiles present changed over time, with aldehydes and alcohols decreased during fruit development in No. 26 by 3 dpa (Fig. 2). In No. 14, aldehyde content decreased from 7.848 μg g^-1^ at 0 dpa to 1.85 μg g^-1^ at 12 dpa, which represented a decrease of 76.43% (Fig. 3). The changes in aldehyde content were insignificant between 9–12 dpa. These results revealed that the aldehyde content decreased rapidly during fruit development, whereas the levels of alcohols and other volatiles were not significantly altered.

### Changes in C6 and C9 volatiles

**Fig 4:**
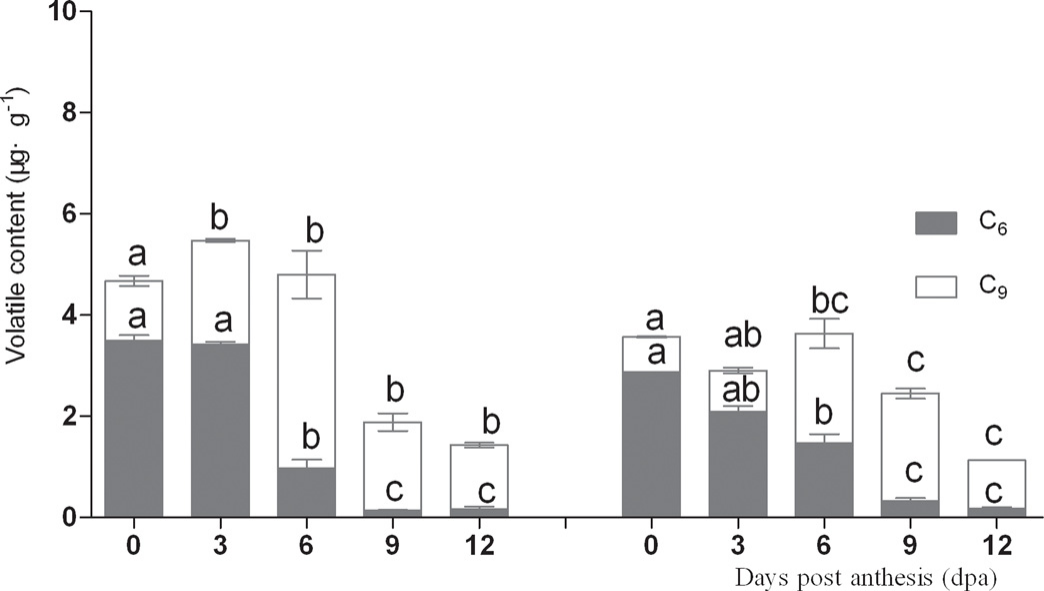
Changes of C6 and C9 content in No. 14 and in No.26 respectively; Left figure: Changes of C6 and C9 content in No. 14; Right figure: Changes of C6 and C9 content in No. 26.

C6 and C9 aldehydes and alcohols were identified as the main compounds in cucumber fruits. C6 aldehydes and alcohols including hexanal, (*E*)-2-hexene, hexanol, (*E*)-2-hexene-1-ol, and (*E*)-3-hexene-1-ol are responsible for the distinctive grassy, green aromas, whereas C9 aldehydes and C9 alcohols including (*E*,*Z*)-2, 6-nonadienal, (*E*)-6-nonena aldehyde, (*Z*)-6-nonene aldehyde, nonanal, (*Z*)-6-nonen-1-ol, (*E*,*Z*)-2,6-nonadien-1-ol, and (*E*,*Z*)-3,6-nonadien-1-ol give cucumber its flower-like aromas. Therefore, these two compound classes have a large influence on cucumber flavor. In this study, C6 aldehydes and alcohols were present at higher concentration during the early stages of fruit development and decreased significantly after 6 dpa, with low concentration detected at 9–12 dpa in both lines (Fig. 4). In contrast, C9 aldehydes and alcohols were present in lower concentration during the early stages of fruit development and increased significantly after 6 dpa, with high concentration at 9–12 dpa in both cultivars.

### Changes in aroma impact compounds

**Fig 5:**
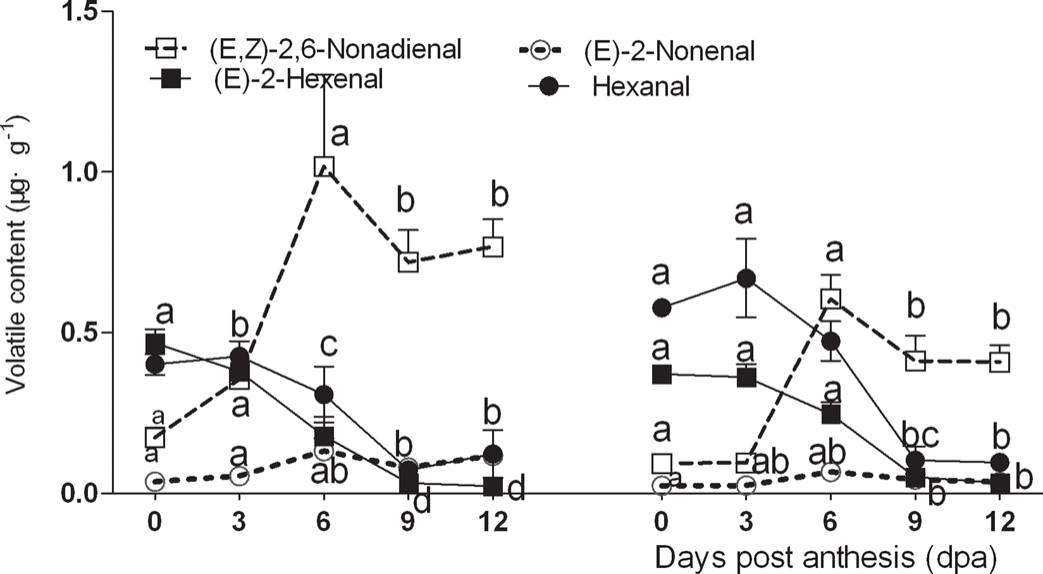
Changes in aroma impact compounds in No. 26 and in No.14 respectively; Left figure: Changes in aroma impact compounds in No. 14; Right figure: Changes in aroma impact compounds in No. 26.

**Fig 6:**
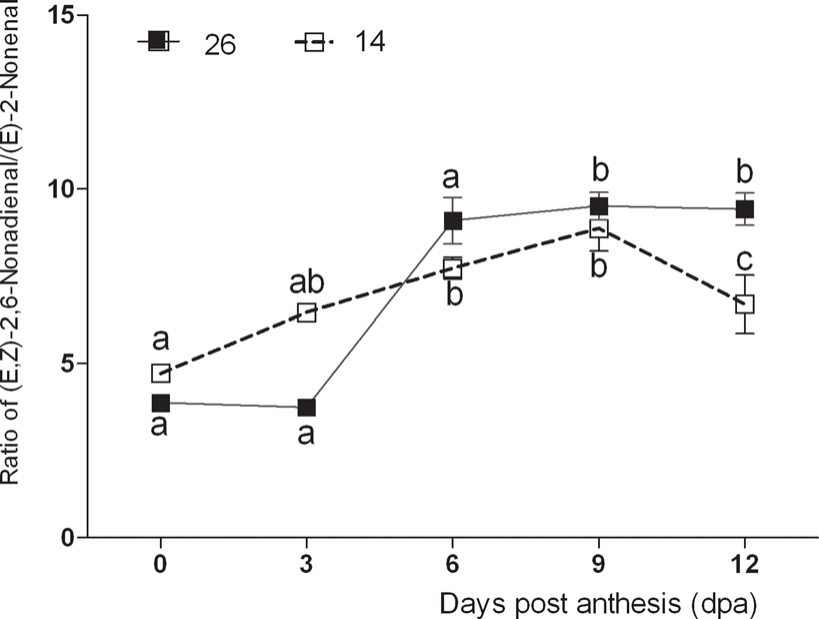
Changes in the (E,Z)-2,6-nonadienal / (E)-2-nonenal ratio.

Both (*E*, *Z*)-2,6-nonadienal and (*E*)-2-nonenal were identified as aroma impact compounds in cucumber fruits. The results revealed that (*E*, *Z*)-2,6-nonadienal first increased and then decreased during fruit development (Fig. 5), with levels peaking at 6 dpa in No.14. In contrast, (*E*)-2-nonenal content only changed slightly during fruit development. (*E*,*Z*)-2,6-nonadienal levels peaked at 3 dpa in No.26, and changes in (*E*)-2-nonenal levels were also subtle in this line. Both (*E*)-2-hexenal and hexanal decreased significantly at the beginning of fruit development, and changed less rapidly between 9–12 dpa in both lines (Fig. 5). The increase in (*E*,*Z*)-2,6-nonadienal and (*E*)-2-nonenal and decrease in (*E*)-2-hexenal and hexanal during fruit development saw ‘green notes’ replaced with ‘cucumber-like’ flavors after 6 dpa. The higher the (*E*,*Z*)-2,6-nonadienal / (*E*)-2-nonenal ratio, the stronger are the fresh cucumber-like flavors, and this ratio increased with fruit development in both cultivars, but was higher in No.26 than in No. 14 at 6 dpa (Fig. 6).

### PCA of volatile compounds at different fruit developmental stages

**Fig 7:**
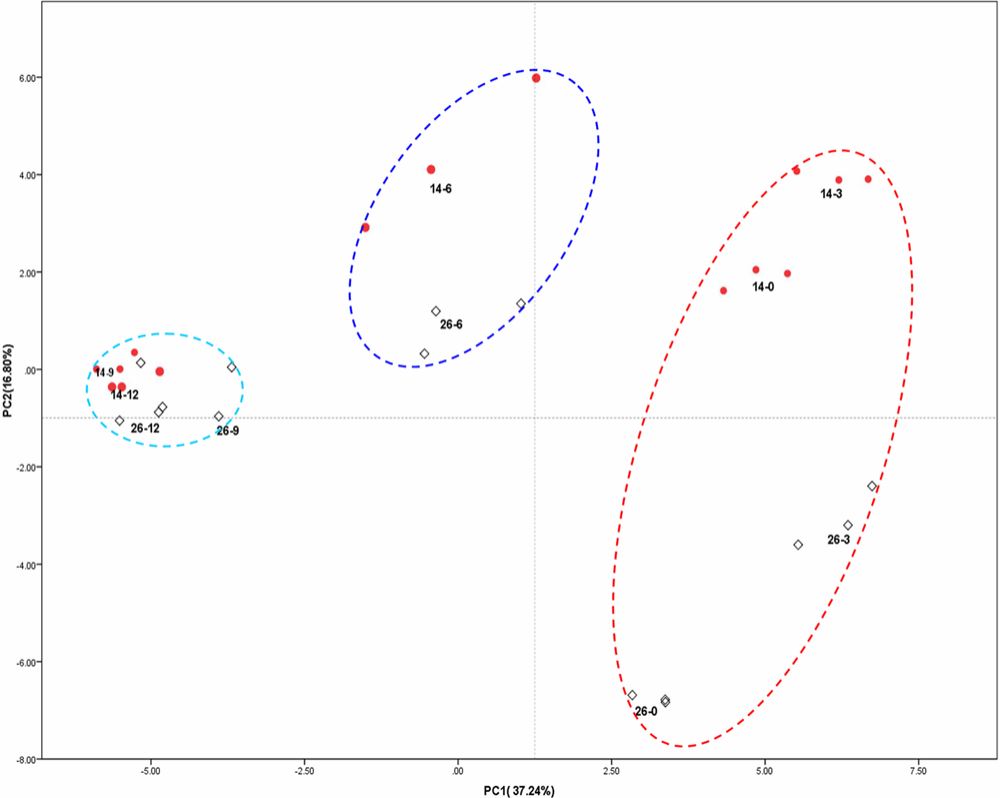
Principal Component analysis (PCA) score plot of volatile compounds during different fruit developmental stages. Round red dots represent volatile compounds from different stages in No.14, and empty rhombuses represent volatile compounds from different stages in No.26. Five independent time course studies, each from triplicate samples, were performed for each time point and used in the analysis, for a total of 10 data points.

PCA allows a large number of variables to be reduced to just a few that accounts for most of the variance in the observed results. We performed PCA to determine which volatile compounds were associated with different fruit developmental stages (Fig. 2). Principal component 1 (PC1) and PC2 accounted for approximately 86.98% of the total variability when volatile compounds were used to characterize cucumber fruit ripening. More specifically, PC1 accounted for 61.11%, and PC2 was responsible for 25.87% (Fig. 7). Five fruit developmental stages were clearly separated from each other, forming distinct clusters (Fig. 7). The 26–0, 14–0, 26–3 and 14–3 stages of the fruit volatile profiles (young fruits for both lines) formed a distinct cluster separated from the other developmental stages. The 26–6, 14–6 stages formed another cluster, as did 26–9, 14–9, 26–12 and 14–12. When volatile compounds were used to characterize the fruit developmental stage, 14–0, 26–0, 14–3, and 26–3 (younger fruits) exhibited the highest similarity in volatile content (Fig. 7), and larger fruits of both cultivars (9–12 dpa) also exhibited similar volatile content during different developmental stages.

**Fig 8:**
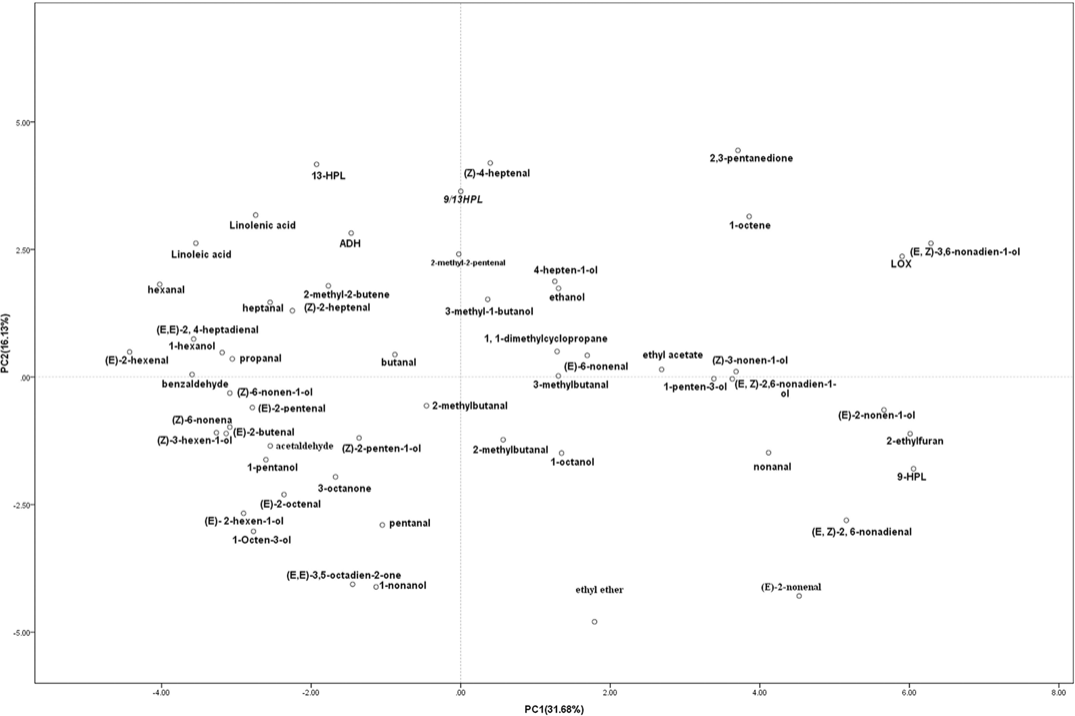
PCA of the different developmental stages based on the first 2 PCA results from volatile compounds.

PCA on the profiles of volatile compounds resulted in a clustered pattern especially for C9, C6 aldehydes and relative alcohols (Fig. 8). C9 aldehydes and relative alcohols were mainly clustered in the positive value of PC1, such as (*E*, *Z*)-2,6-nonadien-1-ol, (*E*)- 6-nonenal, (*E*)-2-nonenal, (E, Z)-2,6-nonadienal, (*Z*)-3-nonen-1-ol, (*E*)-2-nonen-1-ol, (*E*, *Z*)-3,6-nonadien-1-ol, nonanal, while C6 aldehydes and relative alcohols were mainly clustered in the lower negative value of PC1 such as 1-hexanol, (*E*)-2-hexen-1-ol, hexanal, (*Z*)-3-hexen-1-ol, (*E*)-2-hexenal. The higher positive value of PC2 indicated higher 13-HPL enzyme activity, LOX enzyme activity, linoleic acid content, linolenic acid content, and *9/13-HPL* gene relative expression level, higher content of hexanal, 1-hexanol, and (*E*)-2-hexenal, while the lower negative value of PC2 showed higher C9 and associated alcohols (Fig. 8). Most volatiles clustered together and loaded on the negative side of on PC1, whereas the higher aroma impact compound large positive loading on PC1 such as (*E*, *Z*)-2,6-nonadienal and (*E*)-2-nonenal large positive loading on PC1, which suggested a negative correlation with the aroma impact compounds which large negative loading on PC1 such as (*E*)-2-hexenal and hexanal.

Production of C6 compounds such as (*E*)-2-hexenal and hexanal were positively correlated with alcohols such as 1-hexanol, (*Z*)-3-hexen-1-ol, and (*E*)-2-hexen-1-ol; the correlation between (*E*)-2-hexenal and the associated alcohols was positive and highly significant (r = 0.95, 0.90, and 0.65, respectively), and that between hexenal and related alcohols was 0.78, 0.60, and 0.31, respectively. Interestingly, the correlation between C9 aldehydes and associated alcohols was negative and lower. The correlation between (*E*)-6-nonenal and related alcohols 1-nonanol, (*Z*)-3-nonen-1-ol, (*E*)-2-nonen-1-ol, (6*Z*)-nonen-1-ol, (*E*, *Z*)-3,6-nonadien-1-ol, and *E*, *Z*-2,6-nonadien-1-ol was-0.18, -0.17, -0.29, -0.17, -0.05, and -0.33, respectively. The highest correlation found for the (*E*)-2-pentenal was with benzaldehyde (r = 0.98), and very strong correlations were found between the C6 aldehyde (*E*)-2-hexenal and associated alcohols (r = 0.95 and 0.90). The strong correlation between hexanal and related alcohols, and the weaker correlation between C9 and associated alcohols, not only indicated up- regulation or down-regulation of the oxylipin metabolism pathways, but also occurred at different enzymatic steps, which played a key role in biosynthetic pathways of C6 and C9 aldehydes and relative alcohol, such that LOX specially catalysed the production of 9-, 13-, or 9/13 hydroperoxides, and which subsequently were catalyzed by HPL. Furthermore, the hydroperoxide products were catalyzed by ADH to form relative alcohols. The products can be regulated in turn via the amount of substrate, the activity of enzyme, the speciality of the enzyme, and the localization of products along the pathway. This highlighted the question of the relationship among substrate, gene expression, enzyme activity, and speciality of enzyme and the production of these volatiles.

Regulation of the production of C6 and C9 aldehydes during fruit development was apparent at the levels of gene expression and enzyme activity. Production of C6 and related alcohols such as (*E*)-2-hexenal and hexanal were positively correlated with 13-HPL activity and with *CsLOX* and *CsHPL* gene expression, and negatively correlated with LOX and 9-HPL activity. The correlation between (*E*)-2-hexenal and 13-HPL activity was 0.76, and that between hexanal and 13-HPL activity was 0.95, suggesting production of C6 was mainly regulated by 13-HPL activity. Production of C9 aldehydes such as nonenal, (*E*, *Z*)- 2, 6-nonadienal, and (*E*)- 2-nonenal were positively correlated with 9-HPL and LOX activity, but negatively correlated with 13-HPL activity, indicating that production of C9 was mainly regulated by 9-HPL activity. Production of nonenal, (*E*, *Z*)-2,6-nonadienal, and (*E*)-2-nonenal were negatively correlated with *CsLOX* and *CsHPL* gene expression, suggesting other members of the gene family may regulate the production of C9 aldehydes.

### Changes in fatty acid content during fruit development

**Fig 9:**
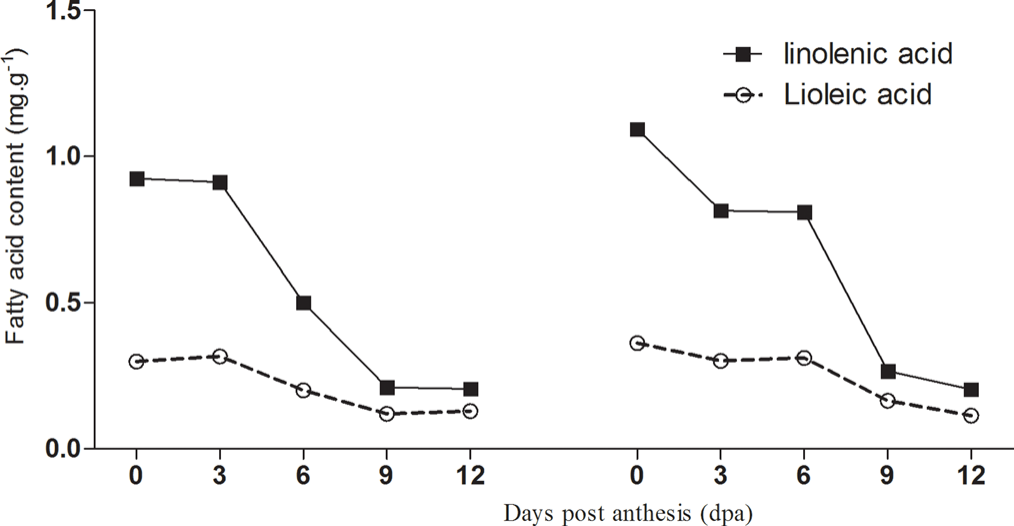
Changes of linolenic acid and linoleic acid content in No. 14 and in No. 26 respectively during cucumber fruit development. Left figure: Changes of linolenic acid and linoleic acid content in No. 14; Right figure: Changes linolenic acid and linoleic acid content in No. 26.

**Fig 10:**
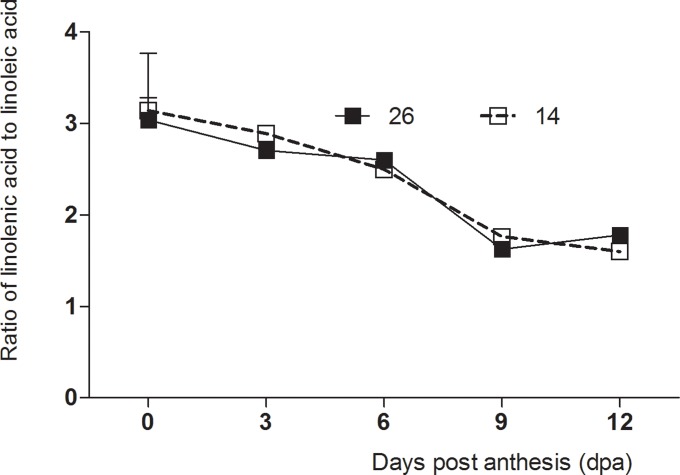
Changes in the linolenic acid / linoleic acid ratio in No. 26 and No. 14 during cucumber fruit development.

Linolenic and linoleic acids are precursors for many volatile compounds, and are the most abundant fatty acids in cucumber fruits [27]. The content of linolenic and linoleic acids changes with fruit development (Fig. 3). The linoleic acid content was highest during the early stages of fruit development, decreased by 3 dpa, and changed only slightly between 9–12 dpa. Overall, fruit development had little influence on linolenic acid content (Fig. 9). Linolenic and linoleic acids are precursors of (*E*)-2-nonenal and (*E*,*Z*)-2,6-nonadienal, respectively. Therefore, the ratio of linolenic to linoleic acid is an important indicator of cucumber fruit flavor. The results revealed that this ratio was highest during the early stages of fruit development, decreased significantly by 6 dpa, and changed only slightly between 9–12 dpa (Fig. 10). The (*E*)-2-nonenal / (*E*,*Z*)-2, 6-nonadienal ratio also decreased with fruit development, which was accompanied by an increase in ‘cucumber-like’ flavor.

Precursor and volatile compound content were both significantly higher during the early stages of fruit development, indicating that substrates for the relevant biosynthetic enzymes were available. C6 aldehydes appeared to account for a large proportion of the volatile compounds during the early stages of fruit development, given the high linolenic acid and linoleic acid content, and LOX activity. During fruit development, the C9 aldehyde content increased gradually while C6 aldehydes decreased, which results in the ‘cucumber-like’ flavor, since this is known to be dependent on the ratio between C6 and C9 aldehydes[11,36]. Green, grass-like flavors dominate in young fruits, whereas fresh cucumber-like flavors dominate in mature fruits. Verzera et al. (2011) reported high levels of ‘green leaf’ volatiles such as hexanal in young melon fruits, but these decreased in mature fruits which contained increased ester content. The strong flavor of melon fruits therefore also develops as fruits mature[31]. Consistent with the results of this study, the ratio of C6 to C9 aldehydes has been previously shown to decrease during cucumber fruit development [42].

### Changes in volatile-associated enzyme activities

**Fig 11:**
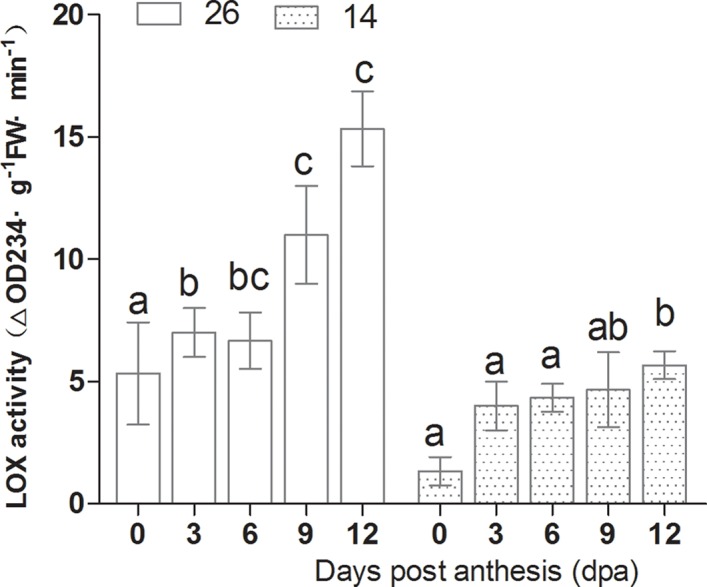
Changes in LOX activity during fruit development.

**Fig 12:**
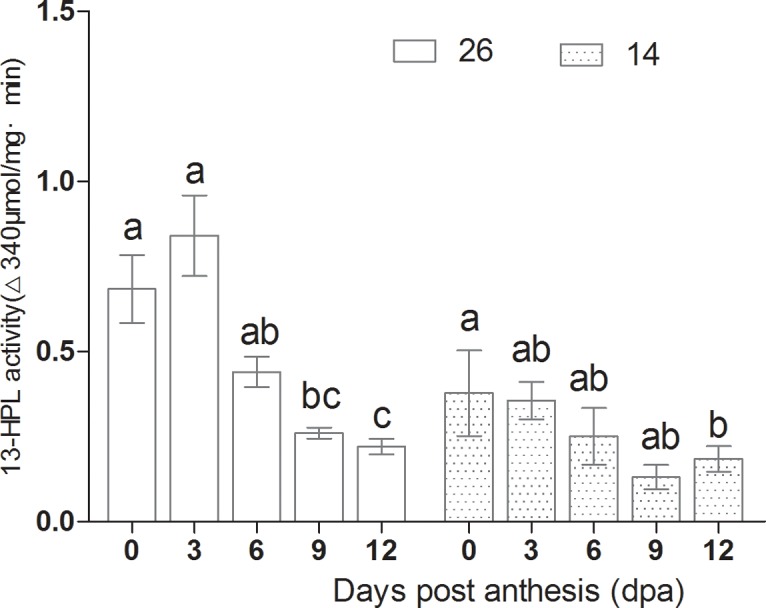
Changes in 13-HPL activity during fruit development.

**Fig 13:**
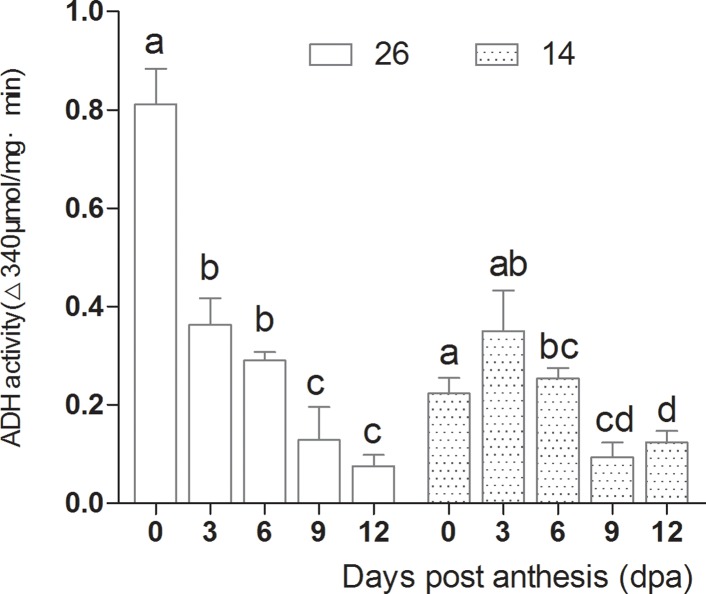
Changes in 9-HPL activity during fruit development.

**Fig 14:**
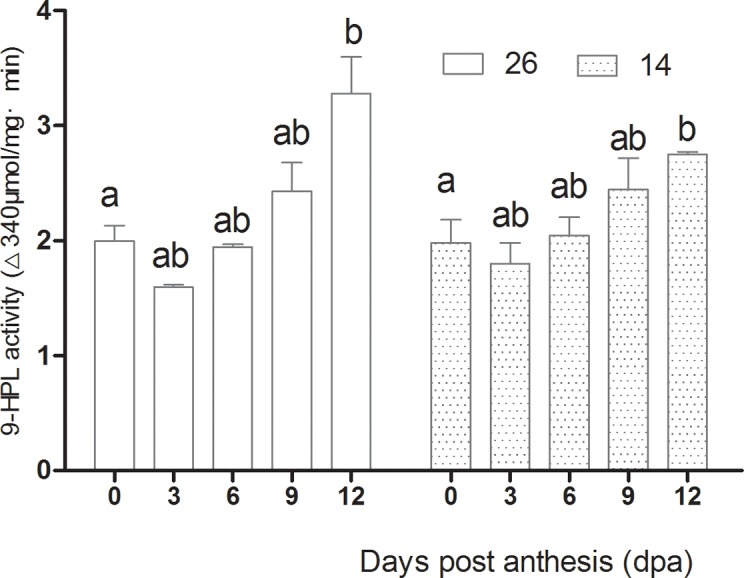
Changes in ADH activity.

LOX activity increased gradually between 0 to 12 dpa, and peaked at 12 dpa, at levels twice those at 0 dpa (Fig. 11). In contrast, 13-HPL and ADH activity were highest during the early stages, decreased gradually during fruit development, were at their lowest at 9 dpa, and changed only slightly from 9–12 dpa (Fig. 12 and Fig. 13). 9-HPL activity increased during fruit development (Fig. 14). The changes in enzyme activity were consistent with the changes in aldehyde and alcohol content during fruit development; ADH and 13-HPL activities were higher between 0–6 dpa, as was the aldehyde and alcohol content.

### Expression of LOX and HPL

**Fig 15:**
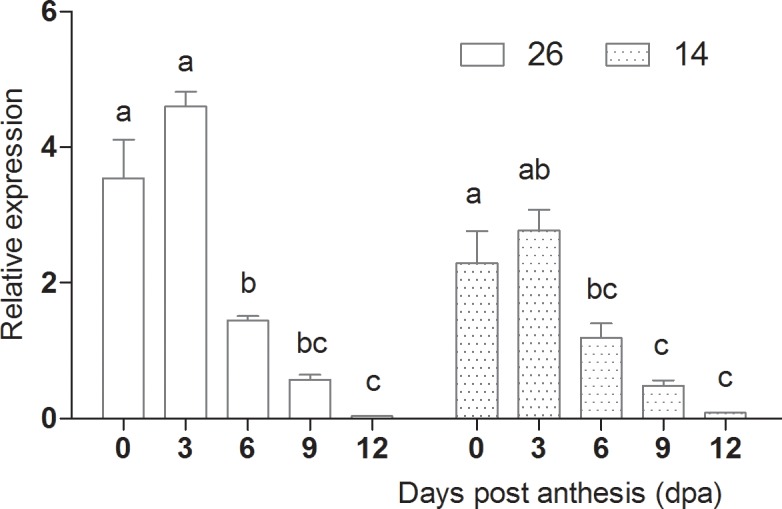
Changes in *LOX* expression during fruit development.

**Fig 16:**
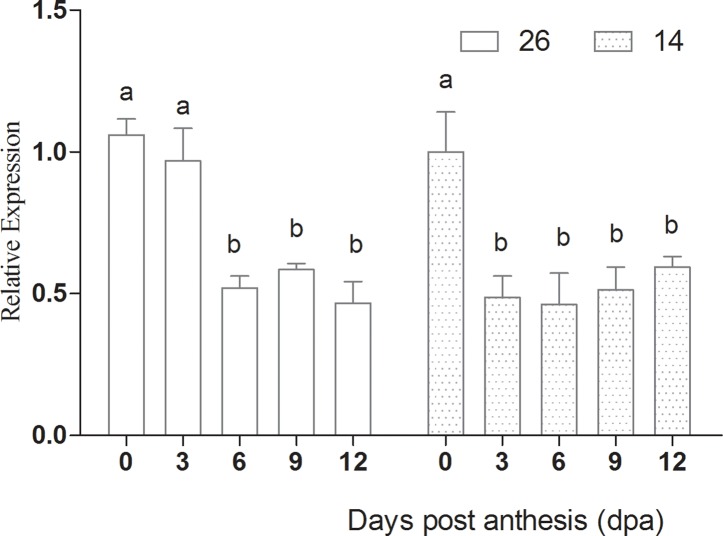
Changes in *HPL* expression during fruit development.

Cucumber flavor is an important indicator of fruit quality. *CsLOX* expression peaked at 3 dpa, and was lowest at 12 dpa, indicating that expression was highest during the early stages of fruit development (Fig. 15). *Interestingly*, *CsHPL* expression differed between the two inbred lines. *CsHPL* expression in No. 26 was highest at 0 dpa and lowest at 12 dpa, suggesting higher levels during the early stages of fruit development. *CsHPL* expression in No.14 was highest at 0 dpa, decreased significantly at 3 dpa, and increased again after 3 dpa. These differences in *CsHPL* expression may be due to differences in genotype (Fig. 16).

### Relationship between aldehyde and fatty acid content, gene expression, and enzyme activity

Aldehydes are the most important compounds in cucumber fruit aroma both quantitatively and qualitatively, and C6 and C9 straight-chain aldehydes are the major contributors. These compounds are produced from polyunsaturated fatty acids such as linoleic and linolenic acids, which are acted on by LOXs and HPLs to produce aldehydes and oxoacids. LOX generates the corresponding 13-hydroperoxide derivatives, and these are subsequently cleaved by HPLs into C6 aldehydes, while 9-hydroperoxides are substrates for production of C9 aldehydes[3,43].

Both *LOX* and *HPL* mediate hydroperoxidation of polyunsaturated fatty acids, leading to the production of n-hexanal and (*E*)-2-hexenal, and these enzymes are important regulators of volatile production in cucumber[44], *Arabidopsis thaliana*[45], tomato [46], lemon[47] and olive [43]. It was reported that specific downregulation of *TomLoxC* resulted in a significant reduction in the levels of C6 aldehydes [46], and HPL activity has also been associated with flavor compound generation in cucumber [36], olive [43] and kiwi [2]. Our current and previous results also found that expression of *CsLOX* and *CsHPL* was higher during the early stages of fruit development and subsequently declined. The *CsLOX* expression profile was very similar in both inbred lines, whereas *CsHPL* expression differed. In cultivar No. 26, *CsHPL* expression was the highest at 0 dpa and lowest at 12 dpa, whereas expression was highest at 0 dpa and lowest at 6 dpa in No. 14, before levels increased again by 12 dpa. *CsLOX* and *CsHPL* expression are therefore dependent on the fruit developmental stage and genotype.

LOX activity increased gradually from 0 dpa and peaked at 12 dpa, in agreement with previous results[11], and Xu et al. (2009) also reported that LOX activity increased gradually from 0 dpa [27].13-HPL activity was found to be higher at 0–6 dpa before decreasing to the lowest levels at 12 dpa in both inbred lines. In contrast, 9-HPL activity was lower at 0–6 dpa and subsequently increased, peaking at 12 dpa.

Maximum expression of *CsLOX* was observed at 3 dpa, whereas maximum LOX activity was measured at 12 dpa, and maximum expression of *CsLOX* therefore preceded maximum LOX activity in both inbred lines. These results may indicate inconsistencies in the expression and enzyme activity data, however similar results were reported for olive [46] and mango [35]. Yang et al. (2012) reported that twelve LOX genes were differentially expressed during fruit development, in which four (*CsLOX1*, *CsLOX4*, *CsLOX8*, and *CsLOX10*) were expressed at levels comparable to *CsLOX*, whereas expression of *CsLOX9* and *CsLOX3* increased during fruit development [44]. Siedow et al. (1991) reported a higher LOX content in mature fruit than in immature fruit, which resulted in higher levels of enzymatic activity [48].

In cultivar No. 26, the expression profile of *CsHPL* was correlated with 13-HPL enzyme activity, although expression peaked slightly earlier, whereas in No. 14 fruits, *CsHPL* expression was correlated with 9-HPL enzyme activity. 13-HPL enzyme activity decreased significantly with fruit ripening in both lines, while LOX and 9-HPL activities increased significantly, which accounted for the higher C6 aldehyde content at 0–6 d dpa, and the higher C9 aldehyde content at 9–12 dpa. LOX and HPL both participate in aldehyde production, and future studies should evaluate the mechanisms by which aldehydes are produced in order to further understand the formation of volatile compounds during fruit development.

**Fig 17:**
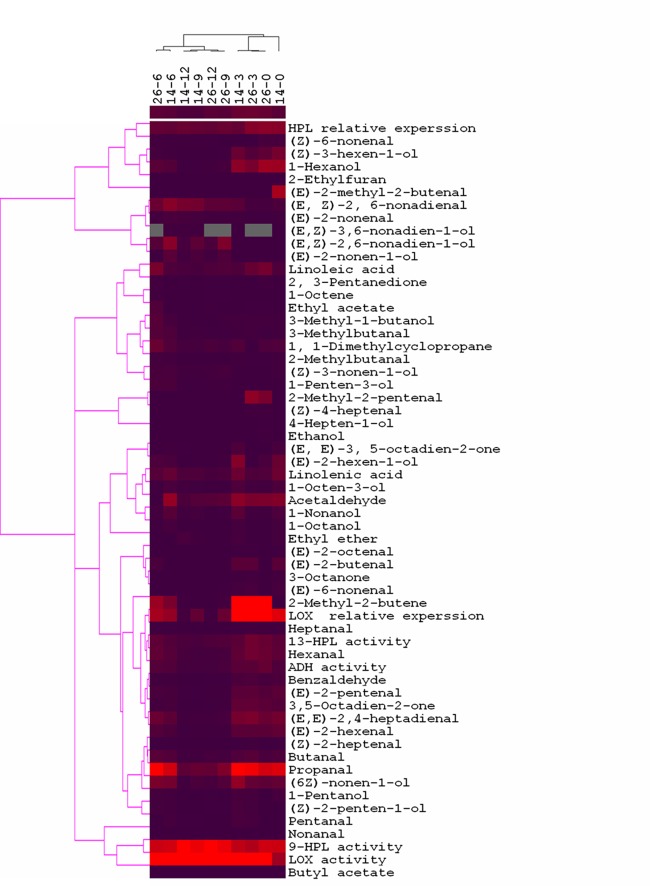
Hierarchical cluster dendrogram and heatmap of volatiles linolenic acid and linoleic acid content, gene expression, and enzyme activity during fruit development in both lines. The stage of fruit development is shown in columns, and volatile compound, linolenic acid and linoleic acid content, gene expression, and enzyme activity are shown in rows. Bright red: high content and high expression; deep purple: low expression; grey: no expression; 14–0~14–12 refer to the five developmental stages of fruits in No. 14 lines, and 26–0~26–12 refer to the five developmental stages of fruits in No. 26 lines.

HCA was employed to explore potential correlations between volatiles, gene expression, and enzyme activity. To stratify samples based on trait, Ward's linkages in Euclidian distance were used. HCA data resulted in two distinct sample clusters (Fig. 17). Cluster I included developmental stages from 6~12 dpa, and displayed the highest 9-HPL and LOX activities and (*E*,*Z*)-2,6-nonadienal, (*E*,*Z*)-3,6-nonadien-1-ol and (*E*, *Z*)-2,6-nonadien-1-ol content (Fig. 17). The (*E*,*Z*)-2,6-nonadien-1-ol content was higher in No. 26 than in No. 14 in cluster I, which contained little (*Z*)-3-hexen-1-ol or 1-hexenol (Fig. 17). Cluster II included developmental stages from 0~3 dpa, and was characterized by higher Cs*HPL* expression and (*Z*)-3-hexen-1-ol and 1-hexenol levels, as well as higher Cs*LOX* expression and acetaldehyde, (*Z*)-2-hexenal, (*E*,*E*)-2,4-heptadienal, hexanal, and ADH activity. In addition, two cultures (cluster 3) grouped together within cluster 1 (Fig. 17), and the first subgroup containing 26–6 and 14–6 shared some characteristics with cluster II.

### Conclusions

These results suggest that the total volatile, C6 aldehyde, linolenic and linoleic acid content, and the (*E*)-2-nonenal / (*E*, *Z*)-2,6-nonadienal ratio were all higher during the stages of fruit development. These parameters then declined, and the C9 aldehyde, hexanal, and (*E*)-2-hexenal content, and the linolenic acid / linoleic acid ratio, that were all low during the early stages, then increased in both lines. Changes in *CsHPL* expression were correlated with 13-HPL enzyme activity in No. 26 fruits, although gene expression slightly preceded 13-HPL activity. In contrast, changes in *CsHPL* expression in No. 14 fruits were correlated with 9-HPL enzyme activity. *CsHPL* expression was therefore associated with 13-HPL activity in cultivar No. 26 from Northern China, but was correlated with 9-HPL activity in cultivar No. 14 from Southern China. It indicated that CsHPL enzyme activity is affected by the ecological setting, as well as the genetic background. 13-HPL enzyme activity decreased significantly with fruit ripening in both lines, whereas LOX and 9-HPL enzyme activities increased significantly, which accounted for the higher proportion of C6 aldehydes at 0–6 dpa and the higher C9 aldehyde content at 9–12 dpa.

## Supporting Information

S1 Table Volatile compounds and contents (μg/g) during cucumber fruit development. (PDF)
