# Supplementary material for: Profiling of Volatile Compounds and Associated Gene Expression and Enzyme Activity during Fruit Development in Two Cucumber Cultivars
Source: PLoS One. 2015 Mar 23;10(3):e0119444. doi: 10.1371/journal.pone.0119444 (PMC4370779; doi:10.1371/journal.pone.0119444)
Supplement: S1 Table — (PDF) [file pone.0119444.s001.PDF]

Table 1. Volatile compounds and contents (μg/g) during cucumber fruit development

Compounds		No. 14					No. 26				
		0 d	3 d	6 d	9 d	12 d	0 d	3 d	6 d	9 d	12 d
Aldehyde	Acetaldehyde	0.929 ± 0.034	1.076 ± 0.121	1.196 ± 0.349	0.293 ± 0.035	0.164 ± 0.055	0.847 ± 0.150	0.820 ± 0.180	-	0.396 ± 0.045	0.299 ± 0.045
	Propanal	2.232 ± 0.158	2.732 ± 0.283	2.076 ± 0.822	0.553 ± 0.092	0.417 ± 0.161	2.091 ± 0.336	2.544 ± 0.531	2.686 ± 0.662	0.895 ± 0.209	0.485 ± 0.136
	Butanal	0.205 ± 0.023	0.228 ± 0.032	0.256 ± 0.082	0.044 ± 0.007	0.046 ± 0.015	0.156 ± 0.034	0.335 ± 0.062	0.346 ± 0.052	0.082 ± 0.011	0.104 ± 0.015
	2-methylbutanal	0.037 ± 0.003	0.031 ± 0.004	0.063 ± 0.017	0.011 ± 0.001	-	0.018 ± 0.004	0.024 ± 0.003	0.072 ± 0.018	0.009 ± 0.001	-
	3-methylbutanal	0.029 ± 0.004	0.040 ± 0.006	0.206 ± 0.075	0.033 ± 0.002	0.007 ± 0.002	0.022 ± 0.006	0.065 ± 0.011	0.296 ± 0.086	0.028 ± 0.004	0.019 ± 0.002
	Pentanal	0.000 ± 0.000	0.132 ± 0.018	0.085 ± 0.026	0.017 ± 0.003	0.027 ± 0.016	-	0.109 ± 0.026	-	-	-
	(*E*)-2-butenal	0.351 ± 0.033	0.361 ± 0.027	-	0.006 ± 0.001	-	-	0.354 ± 0.035	0.148 ± 0.019	0.014 ± 0.003	0.008 ± 0.001
	Hexanal	0.402 ± 0.023	0.427 ± 0.033	0.308 ± 0.071	0.074 ± 0.017	0.123 ± 0.061	0.578 ± 0.005	0.670 ± 0.087	0.474 ± 0.050	0.104 ± 0.035	0.097 ± 0.017
	2-methyl-2-butenal	1.456 ± 0.123	-	-	0.005 ± 0.001	-	-	-	-	-	0.004 ± 0.001
	(*E*)-2-pentenal	0.375 ± 0.026	0.344 ± 0.020	0.118 ± 0.035	0.020 ± 0.006	0.020 ± 0.007	0.271 ± 0.025	0.382 ± 0.035	0.145 ± 0.026	0.029 ± 0.007	0.031 ± 0.004
	Heptanal	0.032 ± 0.002	0.028 ± 0.003	0.015 ± 0.001	0.006 ± 0.001	0.009 ± 0.003	0.044 ± 0.001	0.060 ± 0.004	0.023 ± 0.003	0.008 ± 0.001	0.010 ± 0.001
	(*E*)-2-hexenal	0.468 ± 0.030	0.379 ± 0.007	0.179 ± 0.049	0.033 ± 0.006	0.024 ± 0.007	0.372 ± 0.006	0.362 ± 0.029	0.247 ± 0.031	0.052 ± 0.013	0.033 ± 0.005
	(*Z*)-2-heptenal	0.017 ± 0.004	0.022 ± 0.004	0.027 ± 0.018	0.004 ± 0.001	0.008 ± 0.005	0.014 ± 0.003	0.029 ± 0.008	0.020 ± 0.006	0.005 ± 0.002	0.004 ± 0.000
	(*Z*)-4-heptenal	-	-	-	-	-	0.056 ± 0.003	0.051 ± 0.003	-	-	-
	Nonanal	-	-	-	0.003 ± 0.000	-	-	-	-	-	0.002 ± 0.000
	(*E*)-2-octenal	0.019 ± 0.002	0.021 ± 0.001	0.011 ± 0.004	-	0.006 ± 0.002	-	0.027 ± 0.002	-	-	-
	(*Z*)-6-nonena	0.112 ± 0.005	0.090 ± 0.004	-	-	-	-	0.139 ± 0.002	-	0.017 ± 0.003	-
	(*E*)- 6-nonenal	0.112 ± 0.005	-	0.033 ± 0.023	0.056 ± 0.004	0.049 ± 0.006	0.122 ± 0.002	-	0.062 ± 0.016	-	0.038 ± 0.003
	(*E*)- 2-nonenal	0.037 ± 0.005	0.055 ± 0.001	0.132 ± 0.032	0.081 ± 0.007	0.117 ± 0.023	0.024 ± 0.001	0.026 ± 0.002	0.067 ± 0.009	0.043 ± 0.008	0.040 ± 0.003
	(E,E)-2,4-heptadienal	0.699 ± 0.031	0.767 ± 0.000	0.387 ± 0.138	0.057 ± 0.016	0.051 ± 0.019	0.575 ± 0.007	0.843 ± 0.007	0.607 ± 0.097	0.111 ± 0.030	0.071 ± 0.012
	Benzaldehyde	0.162 ± 0.019	0.184 ± 0.009	0.044 ± 0.015	0.010 ± 0.001	0.014 ± 0.004	0.124 ± 0.001	0.209 ± 0.003	0.049 ± 0.011	0.017 ± 0.005	0.015 ± 0.003
	(*E*, *Z*)-2,6-nonadienal	0.175 ± 0.022	0.355 ± 0.002	1.018 ± 0.232	0.720 ± 0.082	0.768 ± 0.070	0.093 ± 0.002	0.095 ± 0.006	0.605 ± 0.062	0.412 ± 0.064	0.410 ± 0.036
	2-methyl-2-pentenal	-	-	-	-	-	0.807 ± 0.019	1.135 ± 0.144	-	-	-
Alcohols	ethanol	-	-	-	-	-	0.054 ± 0.010	-	-	-	-
	1-penten-3-ol	-	-	0.170 ± 0.057	0.025 ± 0.004	0.026 ± 0.010	-	-	0.173 ± 0.063	0.053 ± 0.013	0.039 ± 0.005
	3-methyl-1-butanol	0.049 ± 0.001	0.060 ± 0.007	0.099 ± 0.025	-	-	0.059 ± 0.007	0.071 ± 0.009	0.334 ± 0.133	0.039 ± 0.002	-
	1-pentanol	0.113 ± 0.005	0.123 ± 0.010	0.070 ± 0.020	-	0.011 ± 0.005	-	0.099 ± 0.006	0.072 ± 0.027	-	0.016 ± 0.001
	(*Z*)-2-penten-1-ol	-	0.138 ± 0.001	0.067 ± 0.020	0.011 ± 0.002	0.011 ± 0.003	-	0.078 ± 0.002	0.072 ± 0.023	0.021 ± 0.004	0.018 ± 0.001
	1-hexanol	1.298 ± 0.045	1.073 ± 0.005	0.281 ± 0.071	0.026 ± 0.003	0.019 ± 0.004	1.338 ± 0.009	0.792 ± 0.008	0.357 ± 0.078	0.091 ± 0.020	0.044 ± 0.004
	(*Z*)-3-hexen-1-ol	0.760 ± 0.020	0.585 ± 0.027	0.052 ± 0.013	-	-	0.439 ± 0.011	0.274 ± 0.000	0.073 ± 0.023	0.019 ± 0.003	-
	(*E*)-2-hexen-1-ol	0.571 ± 0.016	0.961 ± 0.029	0.150 ± 0.037	-	-	0.147 ± 0.009	-	0.191 ± 0.062	0.038 ± 0.007	-
	1-octen-3-ol	0.180 ± 0.012	0.207 ± 0.016	0.129 ± 0.066	0.017 ± 0.003	0.032 ± 0.017	0.043 ± 0.001	0.071 ± 0.006	0.028 ± 0.004	0.008 ± 0.002	-
	4-hepten-1-ol		-	-	-	-	0.022 ± 0.000	-	-	-	-

AcroPDF - A Quality PDF Writer and PDF Converter to create PDF. To remove the line, buy a license.

		No. 14					No. 26				
		0d	3d	6d	9d	12d	0d	3d	6d	9d	12d
	1-octanol	0.082 ± 0.004	0.073 ± 0.006	0.110 ± 0.040	-	-	-	-	0.021 ± 0.006	0.007 ± 0.001	0.007 ± 0.001
	1-nonanol	0.194 ± 0.018	0.383 ± 0.024	0.332 ± 0.045	0.073 ± 0.012	0.030 ± 0.004	0.031 ± 0.001	0.046 ± 0.006	0.057 ± 0.006	0.022 ± 0.002	0.011 ± 0.002
	(*Z*)-3-nonen-1-ol	0.032 ± 0.032	-	0.130 ± 0.035	0.036 ± 0.008	0.041 ± 0.003	-	-	0.114 ± 0.027	0.105 ± 0.008	0.057 ± 0.003
	(*E*)-2-nonen-1-ol	-	-	0.354 ± 0.042	0.180 ± 0.040	0.061 ± 0.012	-	-	-	0.216 ± 0.006	0.052 ± 0.005
	(*Z*)-6-nonen-1-ol	0.506 ± 0.016	1.160 ± 0.002	0.738 ± 0.120	0.157 ± 0.034	0.041 ± 0.005	0.419 ± 0.016	0.498 ± 0.043	0.839 ± 0.167	0.265 ± 0.012	0.111 ± 0.010
	(*E*, *Z*)-3,6-nonadien-1-ol	-	-	0.054 ± 0.008	0.033 ± 0.013	0.042 ± 0.005					
	(*E*, *Z*)-2,6-nonadien-1-ol	-	-	1.032 ± 0.142	0.412 ± 0.100	0.113 ± 0.014	-	-	0.314 ± 0.193	0.876 ± 0.030	0.157 ± 0.014
Ketones	2-ethylfuran	0.013 ± 0.008	0.012 ± 0.007	0.004 ± 0.001	0.002 ± 0.000	0.001 ± 0.000	0.008 ± 0.004	-	0.010 ± 0.002	0.001 ± 0.000	0.003 ± 0.001
	Ethyl acetate	-	-	-	0.005 ± 0.001	0.007 ± 0.002	0.015 ± 0.003	0.018 ± 0.005	0.250 ± 0.067	-	0.037 ± 0.004
	Acetic acid butyl ester	-	-	-	-	-	-	-	-	0.007 ± 0.001	0.007 ± 0.001
	2-methyl-2-butene	-	11.216 ± 1.020	0.823 ± 0.301	-	0.016 ± 0.002	9.017 ± 0.430	10.846 ± 2.253	1.287 ± 0.116	0.067 ± 0.011	-
	1, 1-dimethylcyclopropane	0.235 ± 0.019	0.242 ± 0.014	0.255 ± 0.048	0.083 ± 0.014	0.087 ± 0.023	0.189 ± 0.045	-	0.592 ± 0.198	0.100 ± 0.014	0.169 ± 0.045
	Ethyl ether	0.082 ± 0.018	0.103 ± 0.020	-	0.036 ± 0.005	0.135 ± 0.034	-	-	-	-	-
	1-octene	-	-	-	-	-	-	-	0.053 ± 0.011	0.019 ± 0.005	0.022 ± 0.005
	2,3-pentanedione	-	-	-	0.014 ± 0.002	0.015 ± 0.005	0.055 ± 0.003	-	0.057 ± 0.012	0.022 ± 0.005	0.017 ± 0.001
	3-octanone	0.067 ± 0.006	0.056 ± 0.001	-	-	0.013 ± 0.005	-	0.071 ± 0.008	0.000 ± 0.000	-	0.014 ± 0.002
	(*E*,*E*)-3,5-octadien-2-one	0.403 ±0.005	0.670 ± 0.020	0.168 ± 0.046	0.011 ± 0.002	-	0.370 ± 0.028	0.461 ± 0.006	0.068 ± 0.006	0.040 ± 0.010	0.015 ± 0.003

Note “-” was not detected.

AcroPDF - A Quality PDF Writer and PDF Converter to create PDF. To remove the line, buy a license.
